# Uterine Tumor—Case

**Published:** 1874-08

**Authors:** 


### UTERINE TUMOR—CASE.

By A. COUVERT, M.D.

I report the following case of uterine tumor, it being chiefly interesting from the manner of removing it:

Dr. W. W. Evans, of Oxford, Georgia, gives the following account: "A. K., colored, aged thirty-five, mother of four children; health good up to five years ago, when she miscarried. Since then she has had menorrhagia; sometimes profuse. She has been under frequent treatment by several physicians, with unsatisfactory results. Falling into my hands some time ago, with such symptoms as induced a suspicion of organic lesion, I found, on examination, attached to the inner side of the os, a dense tumor of elongated pear shape, about the size of a turkey egg, extending down into the vagina. A professional friend verified these facts. The patient's health was declining, in spite of treatment, under the almost continuous drain."

We determined on the removal of the offending excrescence. But how to do it was the question, as we had no suitable instruments; ours having gone, like spoons before Butler. We concluded to set our wits to work, and *improvise* some. So, with two reeds, seven inches long, size of pipe-stems, each of exactly equal length, and each wrapped at the end to be introduced, to prevent splitting; next, a flat ring to hold the reeds together; and lastly, a strong flaxen ligature, made like a shoemaker's thread to prevent kinking, and well waxed with beeswax.

Thus equipped, in full harmony with the rude hut of our patient, we proceeded to work. One end of the cord was passed through each reed, and drawn so as to bring them parallel and in contact. They were next introduced up to the neck of the tumor, which was about the size of a man's thumb. One reed was held there, while the other was passed around the tumor, and brought parallel and in contact with its fellow, thus encircling the neck of the tumor with the cord. The ring was then put on and pushed up near the inner ends of the canulæ or reeds; the cord drawn as tightly as possible, tied and wrapped around the outer ends of the canulæ. On the second day an attempt was made to tighten the ligature, but without any perceptible success, so tight was the first ligation made.

We used a large ligature, because we feared cutting the pedicle by a small one, and we wished to apply such force as would strangulate the tumor at once, and thus avoid the septic dangers of delay; and all the more as we found (which we previously expected) that ligation gave no pain. In less than three days the tumor passed off while she was over the vessel for another purpose.

About twenty-four hours after the ligation the stench became almost intolerable; pulse rose to 110; temperature to 102. Fearing septicæmia, we ordered copious detergent vaginal injections, frequently repeated, and followed each time by bromo-chloralum, largely diluted with water, which proved a very efficient antiseptic and deodorizer. As the patient was very feeble, analeptics, both medicinal and dietetic, were freely given. But, not to tax your patience with unnecessary details;, suffice it to say, all adverse symptoms yielded in two or three days. Improvement soon became rapid and decided, until now her health is better than it has been for five years past. Neither the touch nor the speculum reveals any vestige of the tumor or pedicle remaining.

The tumor was fibrous and dense, and tight ligation only could have brought it away so soon, and thus averted the threatened dangers, which, even in skillful and accustomed hands, with appropriate instruments, have so often resulted in death.

Since the foregoing was written Dr. Evans visited the cabin of our patient; but she was not seen, being with her husband out in the field hoeing cotton, with June's sun shining on her, thirty-eight days after the operation.

